# Inflammation and transcriptional responses of peripheral blood mononuclear cells in classic ataxia telangiectasia

**DOI:** 10.1371/journal.pone.0209496

**Published:** 2018-12-26

**Authors:** Sharon A. McGrath-Morrow, Roland Ndeh, Joseph M. Collaco, Cynthia Rothblum-Oviatt, Jennifer Wright, Michael A. O’Reilly, Benjamin D. Singer, Howard M. Lederman

**Affiliations:** 1 Eudowood Division of Pediatric Respiratory Sciences, Johns Hopkins School of Medicine, Baltimore, Maryland, United States of America; 2 A-T Children’s Project, Coconut Creek, Florida, United States of America; 3 Eudowood Division of Pediatric, Allergy and Immunology, Johns Hopkins Medical Institutions, Baltimore, Maryland, United States of America; 4 Department of Pediatrics, School of Medicine and Dentistry, The University of Rochester, Rochester, New York, United States of America; 5 Division of Pulmonary and Critical Care Medicine, Department of Medicine, Northwestern University Feinberg School of Medicine, Chicago, Illinois, United States of America; 6 Department of Biochemistry and Molecular Genetics, Northwestern University Feinberg School of Medicine, Chicago, Illinois, United States of America; 7 Simpson Querrey Center for Epigenetics, Northwestern University Feinberg School of Medicine, Chicago, Illinois, United States of America; Cornell University, UNITED STATES

## Abstract

**Introduction:**

Classic ataxia telangiectasia (A-T) is an autosomal recessive disease characterized by early onset ataxia, immune deficiency, sino-pulmonary disease, lymphoid/solid malignancies and telangiectasias. Prior studies have suggested that chronic inflammation and premature aging may contribute to the development of malignancy and pulmonary disease in people with A-T. To further examine the link between A-T and inflammation, we hypothesized that subjects with classic A-T would have greater enrichment of inflammatory pathways in peripheral blood mononuclear cells (PBMCs) compared to non A-T age-matched controls. To test this hypothesis we used RNAseq as an unsupervised approach to identify biological processes altered in people with classic A-T.

**Methods:**

PBMCs were isolated from subjects with classic A-T and compared to non-A-T age-matched healthy controls. RNAseq with differential gene expression analyses was then performed. Selected genes were validated by RT-qPCR using cohorts of subjects consisting of classic A-T, mild A-T or non-A-T controls. Subjects with mild A-T were characterized by later onset/mild neurologic features and normal/near normal immune status.

**Results:**

RNAseq revealed 310 differentially expressed genes (DEGs) including genes involved in inflammation, immune regulation, and cancer. Using gene set enrichment analysis, A-T subjects were found to have biological processes enriched for inflammatory and malignancy pathways. In examining a cohort of A-T subjects in which baseline serum IL8 and IL6 levels were measured previously, an association was found between higher serum IL8 levels and higher likelihood of developing malignancy and/or death in a subsequent 4–6 year period.

**Conclusion:**

RNAseq using PBMCs from subjects with classic A-T uncovered differential expression of immune response genes and biological processes associated with inflammation, immune regulation, and cancer. Follow-up of A-T subjects over a 4–6 year period revealed an association between higher baseline serum IL8 levels and malignancy/death. These findings support a role for inflammation as a contributing factor in A-T phenotypes.

## Introduction

Classic ataxia telangiectasia (A-T) is a rare autosomal recessive disease characterized by cerebellar degeneration, ocular telangiectasias, lymphoid/solid malignancies, immune dysregulation and sino-pulmonary disease. Individuals with classic A-T present with early onset ataxia, immune deficiencies and frequent sinopulmonary disease. In contrast, there is a subset of people with mild A-T who present with later onset/mild neurologic symptoms and minimal deficiency or sinopulmonary disease. Individuals with mild A-T, frequently have ATM mutations associated with some ATM kinase activity, [1] which likely contributes to the milder phenotypes observed in these patients. [2] Understanding how biological processes are dysregulated among the different subsets of people with A-T may provide insights for targeting therapies in a more personalized approach.

Chronic inflammation and premature aging are factors that likely contribute to A-T related phenotypes.[3] Aging cells can acquire a senescence-associated secretory phenotype (SASP) associated with chronic inflammation, higher risk of cancer development and neurodegeneration. [4] [5] [6] Upregulation of the pro-inflammatory cytokines, IL6 and IL8 have been associated with the SASP phenotype.[7] IL6 has been associated with poorer cancer outcomes [8] while elevated levels of serum IL8 have been associated with colorectal cancer.[9] We previously reported on a subset of children and adults with A-T who had elevated serum levels of IL6 and IL8. [10] [11] In our studies, we found an association between higher baseline serum levels of IL6 and IL8 and lower lung function. We also found a trend towards an association between higher cytokine levels and having a history of malignancy. To this end, we hypothesized that subjects with classic A-T would have biological processes enriched for inflammatory and malignancy pathways. Also, since inflammation is an identifiable factor associated with malignancy, we were interested in determining if higher serum IL6 and IL8 levels were associated with cancer development in people with A-T over time.

During the first two decades of life, lymphoid cancers are the most common cancers in people with classic A-T. Cancer risk however is also increased in people with mild A-T, with solid cancers becoming more prevalent with increasing age.[12–14] In addition, the approximately 1–2% of adults who are carriers of ATM mutations have a higher risk for developing cancer, particularly cancers of the breast and digestive tract. [15] [16] Determining if a link exists between inflammation and A-T may help identify subsets of people with A-T at higher risk for developing malignancy.

Mitochondrial dysfunction is another factor that may contribute to chronic inflammation in A-T, leading to genotoxic stress, and inflammation. [3] In murine models, NAD^+^ repletion improved mitochondrial dysfunction in neurons of ATM knockout (KO) mice, and nitroxide antioxidant administration reduced oxidative stress, restored mitochondrial membrane potential and prolonged latency in developing thymic lymphomas in ATM KO mice. [17] [18] Finally, the increase risk of cancer with aging is associated with oncogene activation and silencing of tumor suppressor genes through epigenetic changes. [19] Since individuals with A-T have a premature aging phenotype, this could be a factor in the higher malignancy rates in A-T.

In A-T the biological processes that contribute to inflammation, immune dysregulation and cancer have not been clearly elucidated. In this study, PBMCs were isolated and used for RNAseq studies, as an unsupervised approach, to identify differentially expressed genes (DEGs) and biological processes enriched in people with classic A-T. Based on classic A-T phenotypes, we hypothesized that genes involved in inflammatory pathways and immune regulation would be altered in subjects with classic A-T compared to non-A-T healthy controls. Identifying molecular pathways that underlie the inflammatory phenotype in A-T may help with developing targeted therapies needed to modify disease progression.

## Methods

### Subjects

The institutional review board of the Johns Hopkins Medical Institutions approved the study (IRB NA_00051764) and written informed consent was obtained from every participant and/or his/her guardian. All subjects with A-T met the diagnosis of A-T based on clinical symptoms and laboratory findings of either elevated alpha-fetoprotein, diminished ATM protein, and/or increased chromosomal breakage after *in vitro* exposure to x-rays, as previously established. [20] Demographic information was obtained from chart review. The three A-T subjects included in the RNAseq analyses were 13, 18 and 26 years of age and included two males and one female. The age-matched normal controls included in the RNAseq analyses were 12, 23 and 29 years of age and included two males and one female. Separate cohorts of subjects were recruited and used for validation of genes by RT-PCR. These groups included subjects with classic A-T phenotypes (n = 3–7), mild A-T phenotypes (n = 8) and non A-T healthy controls (n = 6). We also re-examined subjects with A-T from a previously published cohort of patients [11] with updated outcome data.

### Pulmonary function testing

Subjects with the diagnosis of A-T underwent standard spirometry according to recommendations by the American Thoracic Society.[21] During each visit a minimum of 3 flow–volume curves were attempted/person (MedGraphics). The best flow volume loop per visit was selected for further evaluation. Wang predicted values were used for children up to 16 years of age, [22] and NHANES III predicted values were used for adolescents over age 16 years [23].

### Peripheral blood mononuclear cell (PBMC)

PBMCs were isolated using the Ficoll-Paque Plus Method, which was designed for *in vitro* isolation of lymphocytes (GE Healthcare Bio-sciences AB). Briefly, the blood sample was transferred into a 50 ml conical tube and PBS was added to achieve a 20ml volume. 15 ml of Ficoll- Paque plus was transferred into a separate 50 ml conical tube using a syringe. The 20 ml of diluted blood was gently layered onto the Ficoll. The sample was spun down at 400xg for 30 minutes at room temperature with no brake. The PBMC layer was then collected at the diluted plasma/ficoll interface. PBS was added to the PBMC to bring the volume up to 20 ml. The sample was then spun down at 200xg for 10 minutes at room temperature. The supernatant was discarded and the cells were resuspended in 1 ml of PBS and counted using a TC20 automatic cell counter from Bio-Rad. 4 ml of PBS was added to the sample and spun down at 200xg for 10 minutes, to remove any contaminating Ficoll and platelets/plasma proteins. For RT-qPCR, The supernatant was discarded and the cells where lysed in 1 ml of trizol and allowed to sit at room temperature for 5 minutes before being transferred to a -80 degrees freezer. For RNAseq, the sample was resuspended in 350ul of RLT plus containing beta-mercapto ethanol, vortexed for 30 seconds and then transferred to a -80 degrees freezer.

### RNA isolation

For RNAseq, RNA was isolated using Qiagen RNeasy Plus Mini kit protocol.

For RT-qPCR, RNA was isolated using trizol/chloroform (abcam) and cDNA was synthesized using iScript cDNA synthesis kit (Biorad).

### RT-qPCR

Reactions were performed in triplicate, using 2ul of template per reaction of cDNA in qPCR master mix, (Applied Biosystems). The Quantitative- Comparative C_T_ (ΔΔC_T_) program on the machine was used for the qPCR. The following taqman primers/probes (ThermoFisher Scientific, Applied Biosystems) were used: ADAM23 (Hs00187022_m1), NRCAM (Hs01031598_m1), CNTNAP2 (Hs01034296_m1), LTBP1 (Hs01558763_m1), ITGB5 (Hs00174435_m1), TAL1 (Hs01097987_m1), CLU (Hs00156548_m1), ALOx12 (Hs00167524_m1), and ADRA2A (Hs01099503_s1). A GAPDH (Hs02786624_g1) internal control assay was used. All the Taqman expression assays had a FAM dye except the GAPDH, which had a VIC dye. Both the internal control gene (GAPDH) and the gene of interest were ran in the same reaction.

### RNA sequencing, processing and analysis

Transcriptional profiling was performed as previously described [24] with the following modifications. Libraries for RNA sequencing were prepared with the Illumina TruSeq Stranded Total RNA library prep kit and sequenced using 100 x 100 paired-end reads on an Illumina HiSeq 2500 instrument. Fastq files were aligned to the hg19 reference genome using rsem v1.2.29, which was also used to generate gene and transcript expression levels. Differential gene expression analysis was performed with edgeR v3.16.5 (R v3.4.3 with RStudio v0.98.1103). Lowly expressed genes were excluded by including only those genes with more than 1 count per million in at least 2 of the samples. Library sizes were re-computed after filtering these genes, and sample normalization factors were calculated and applied using the trimmed mean of M values (TMM) procedure as implemented in edgeR. We then performed a pairwise statistical comparison between control and A-T samples by fitting a generalized linear model and executing a likelihood ratio test followed by false-discovery rate (FDR) correction using the Benjamini-Hochberg method. An FDR q-value cutoff of 0.05 was used to identify differentially expressed genes. Gene set enrichment analysis was performed using the GSEA 3.0 GSEAPreranked tool testing for enrichment of the Hallmark Gene Sets, with genes ordered by log difference in average expression comparing controls versus A-T.

We have included in the Supplemental Data raw processed counts data for the transcriptional profiling studies (S1 Table).

### Generation of heat map and Volcano plots

edgeR analysis using an FDR q-value cutoff of 0.05 was used to identify differentially expressed genes. Lowly expressed genes were excluded by including only those genes with more than 1 count per million in at least 2 of the samples.

### Cytokine analysis

As previously described [11], venous blood was drawn from individuals undergoing outpatient evaluation at the Johns Hopkins A-T clinic between 2012 and 2014. Subjects were at baseline health and were not acutely ill at the time of their blood draws. The sera were separated and frozen at -80 C until analyzed. Concentrations of IL-6 and IL-8 were determined using commercially available EIA kits (R& D Systems, Minneapolis, MN). The optical density of each sample was determined using a microplate reader set to 450nm (Optimax, Molecular Devices, Sunnyvale, CA). Data was calculated from a standard curve and the results reported in pictograms of cytokine protein per milliliter for each cytokine. The mean minimum detectable dose for IL-6 was 0.7 pg/mL and for IL-8 was 3.5 pg/mL. Samples were assayed in duplicate and values were expressed as means +/- SD. All sample testing was performed in a masked fashion.

### Statistics

Associations between demographic and clinical factors were assessed using *t*-tests and multivariable logistic regression modeling. Standard deviations were reported unless otherwise stated. For Log RT-qPCR analysis, *P* values were derived through linear regression and adjusted for age.

## Results

### Subjects demographics

RNAseq was performed on PBMCs obtained from subjects with classic A-T. All A-T subjects met the diagnosis of A-T, based on clinical symptoms and laboratory findings of either elevated alpha-fetoprotein, diminished ATM protein, and/or increased chromosomal breakage after *in vitro* exposure to x-rays as previously established. [20] All A-T subjects had neurological symptoms of ataxia, bulbar conjunctival telangiectasias, abnormal radiosensitivity of lymphocytes, immunodeficiency and elevated α-fetoprotein levels (mean 419.7ng/ml ± 168.6, SEM). Spirometry revealed moderate restrictive lung disease (FVC- 50.3% predicted of normal ± 3.8%, SEM). The mean age of subjects with classic A-T was 19 years ± 6.5 and the mean age of healthy non-A-T controls was 21.3 years ± 8.6 (p<0.728). Both the A-T and non A-T groups included two males and one female.

### RNAseq

**Fig 1:**
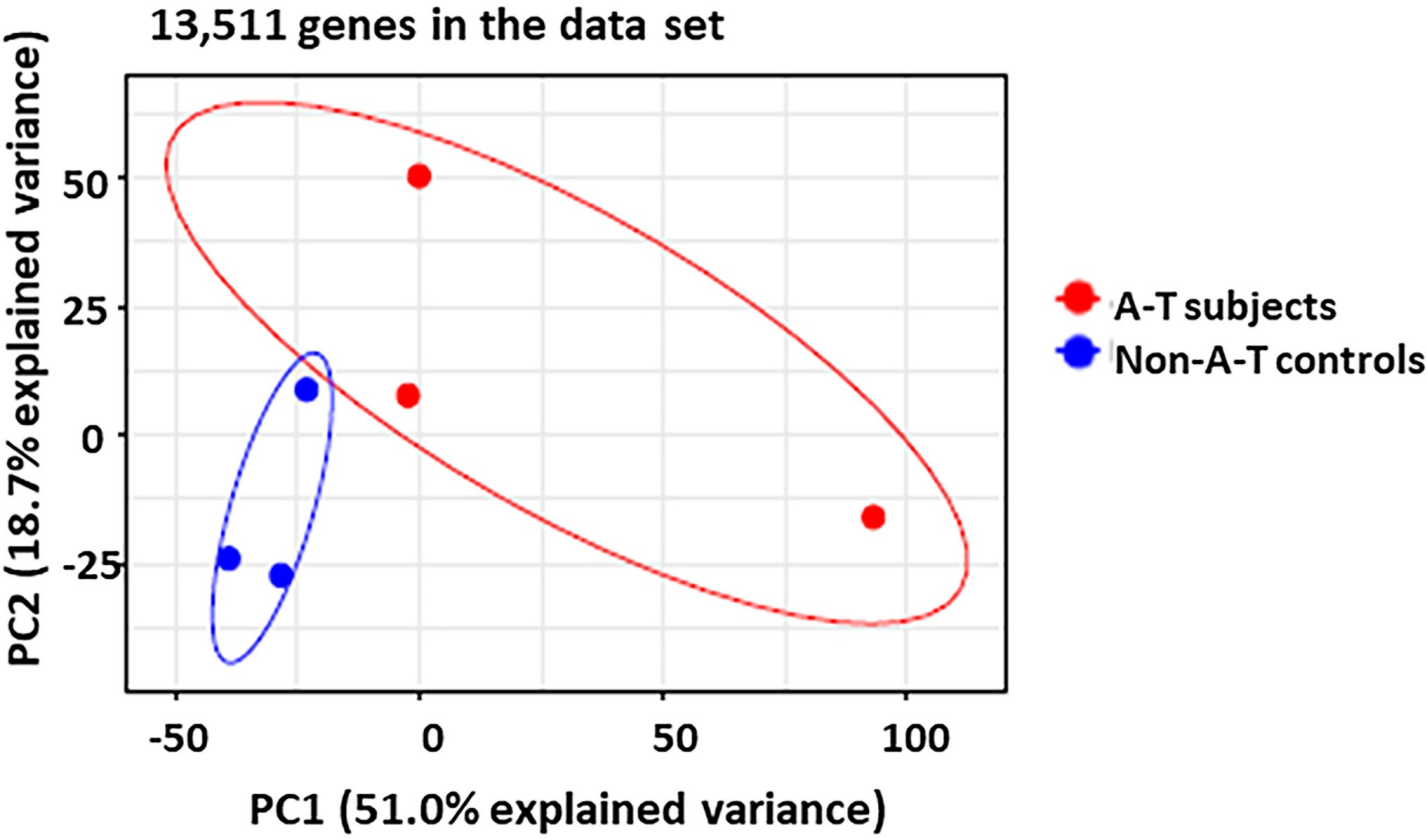
Principal Component analysis (PCA) illustrating variance across 13,511 genes in the data set. Variance is primarily due to classic A-T phenotype (PC1). Red- Classic A-T phenotype (n = 3) and Blue- non-A-T healthy age-matched controls (n = 3). Ellipses represent normal contour lines with one standard deviation probability.

RNAseq was used as an unsupervised approach to identify differentially expressed genes (DEGs) and biological processes altered in subjects with classic A-T. Principal component analysis (PCA) of the RNAseq data set revealed the influence of intergroup variability (classic A-T versus non-A-T control) and intragroup variability (biological and technical variability within replicates in a group). Across the entire data set of 13, 511 genes, PCA showed distinct clustering by group assignment (classic A-T versus non-A-T). (Fig 1) Principal component 1 contained over half of the variance in our study (PC1-51%) and was driven primarily by the classic A-T phenotype.

**Fig 2:**
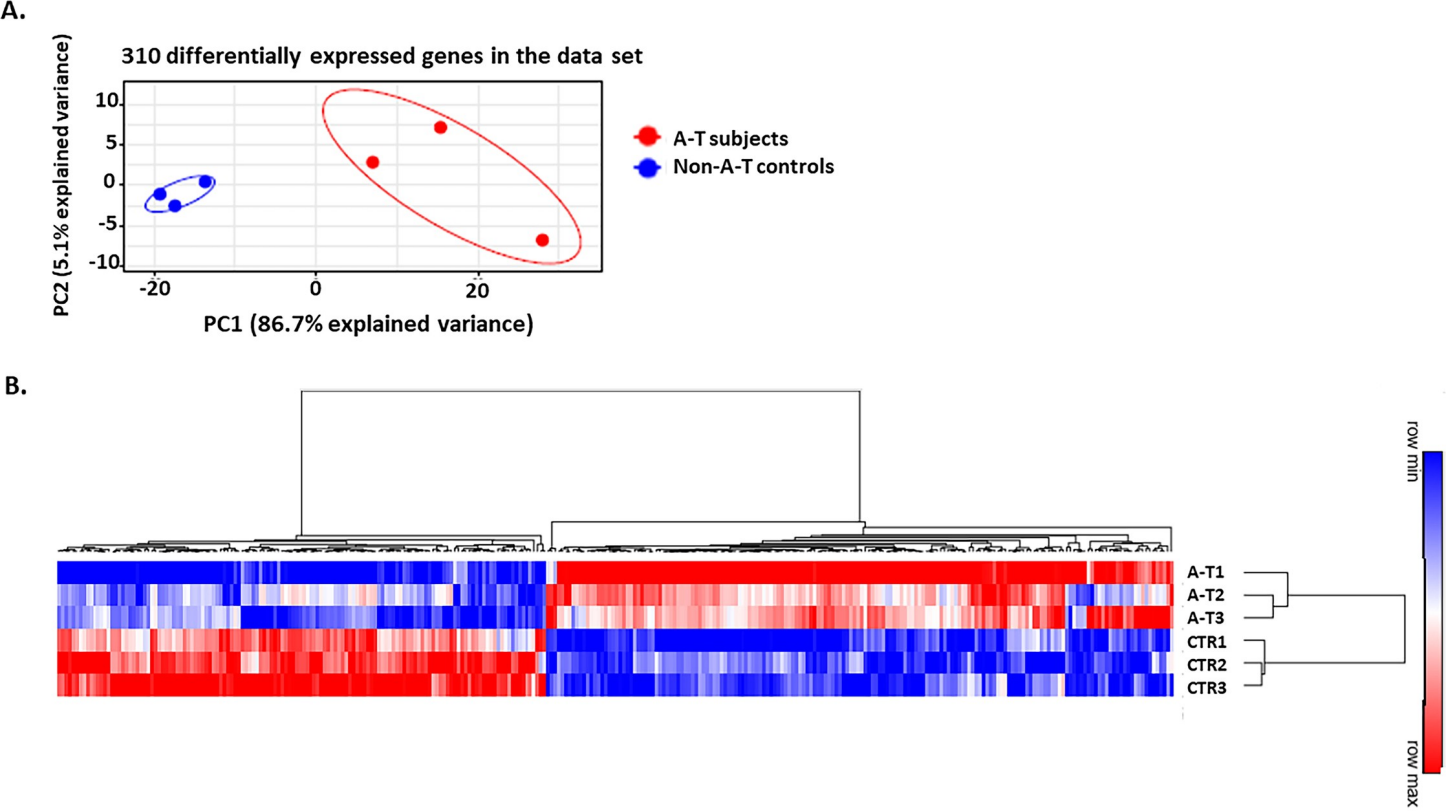
**A**. Principal Component analysis using 310 DEGs identified in the RNAseq dataset. **A** PC1 variance of 86.7% indicates that A-T phenotype accounts for the majority of variance between the two groups. **B.** Heat map of the 310 differentially expressed genes. AT1, AT2, AT3 represents individual samples in the classic A-T group and Ctr1, Ctr2 and Ctr3 represent individual samples in the non-A-T healthy age-matched control group. The rows and columns are grouped using hierarchical clustering. Red- represents increased gene expression and blue represents decreased gene expression. DEGs were identified using edgeR analysis, with a FDR q-value cutoff of 0.05.

Multiple group testing with a false discovery rate (FDR) q-value ≤ 0.05, revealed 310 differentially expressed genes (DEGs) between the two groups. PCA of the DEGs, revealed that explained variance was again primarily due to classic A-T phenotype, (Fig 2A). A heat map of the 310 DEGs grouped according to hierarchical clustering revealed distinct gene expression differences, with clusters of upregulated (red) and downregulated (blue) genes assigned to each group (Fig 2B).

**Fig 3:**
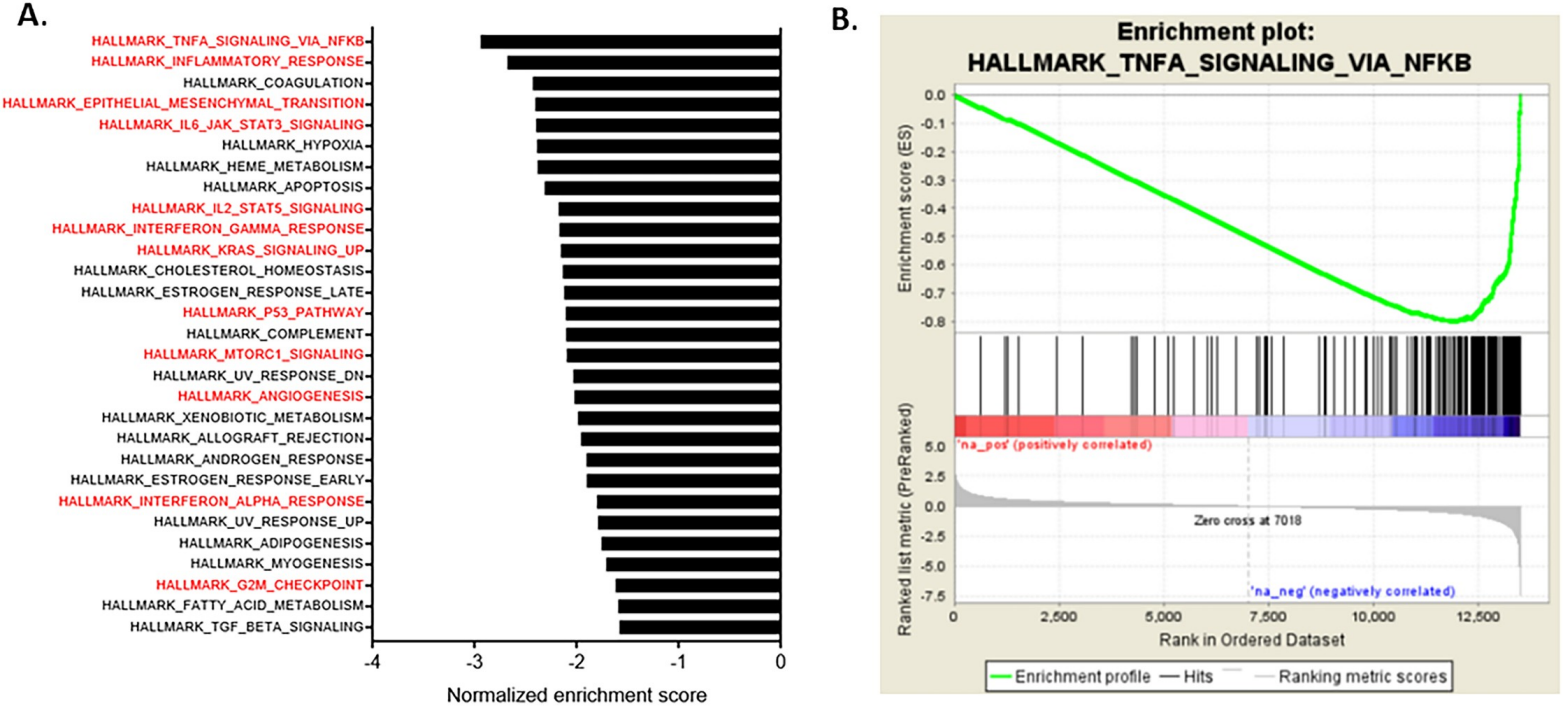
**A.** Hallmark processes negatively enriched in controls (positively enriched in A-T). Attached bar graph includes hallmark processes negatively enriched in non A-T age-matched healthy controls (positively enriched in A-T) with a FDR q-value < = 0.01. Processes highlighted in red are processes involved in inflammation, cancer and cell proliferation. **B.** Example gene set enrichment plot for TNF-α signaling via NFκB.

We next used gene set enrichment analysis [25] to rank hallmark processes negatively enriched in controls and positively enriched in A-T subjects, (Fig 3A). Top biological processes in A-T subjects included those involved in inflammation (TNFα signaling, inflammatory responses, IL6 signaling), immune regulation (IL2, Interferon gamma, Interferon alpha), cancer (KRAS, MTORC1, apoptosis) and cell growth (G2M checkpoint, P53). Fig 3B illustrates an enrichment plot for TNFα signaling via NFKβ, a pathway involved in inflammation. In addition, enrichment of an angiogenesis pathway was also found in subjects with classic A-T, supporting a previous study that reported an association between presence of telangiectasias and ATM deficiency. [26] Enrichment plots for the top 15 processes are included in S1 Fig.

**Fig 4:**
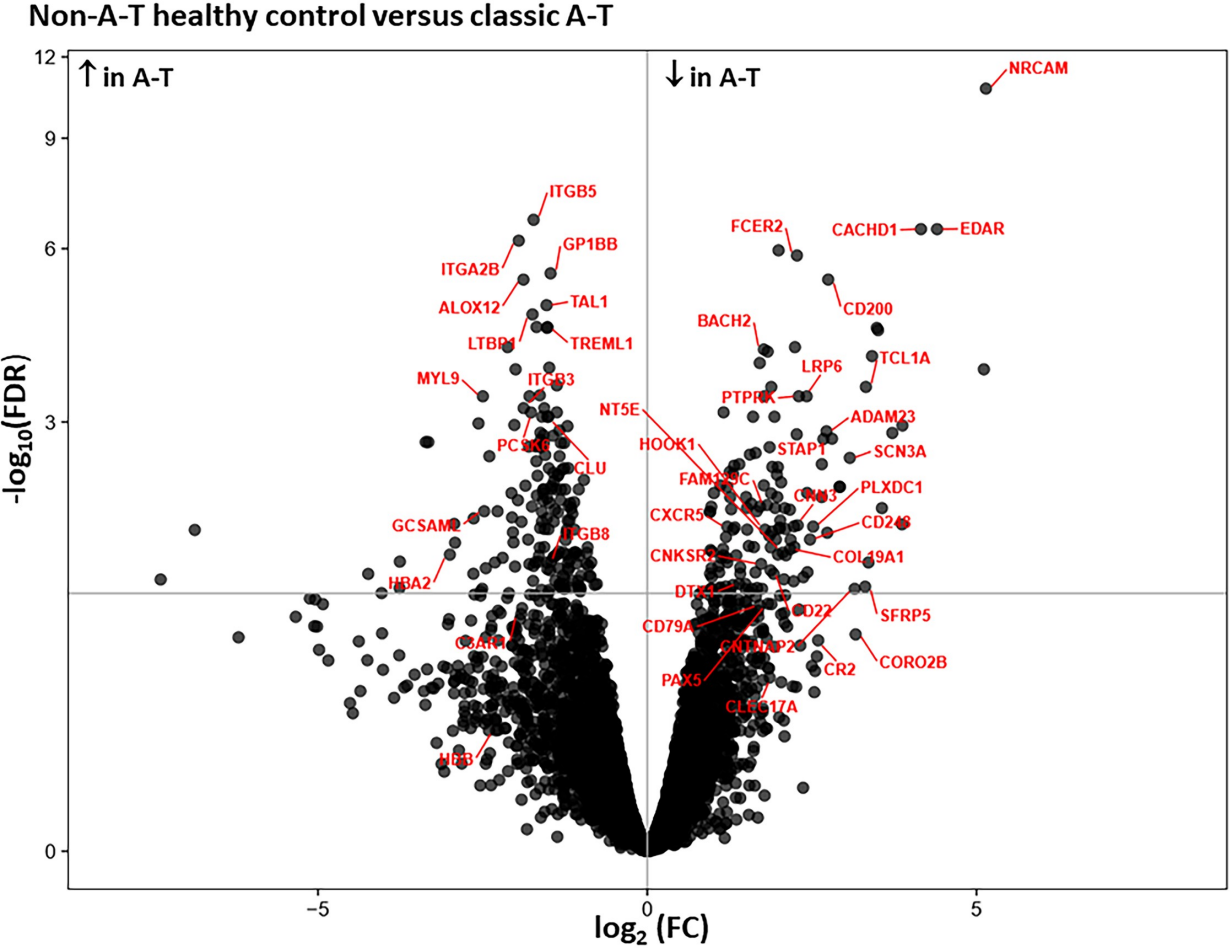
Volcano plot of 310 DEGs depicting genes downregulated in the classic A-T group (right side) and upregulated in the classic A-T group (left side) compared to non-A-T healthy age-matched controls.

Fig 4 illustrates a volcano plot of genes differentially expressed in subjects with A-T compared to age-matched controls. These genes include the immune and tumor suppressor genes, ADAM23 [27] and BACH2 [28] and CD200, a molecule that helps to regulate immune activation. [29] All three of these genes were downregulated in the A-T subjects.

### Serum levels of IL8 in A-T associated with higher risk of malignancy and death over a 4–6 year follow-up period

**Fig 5:**
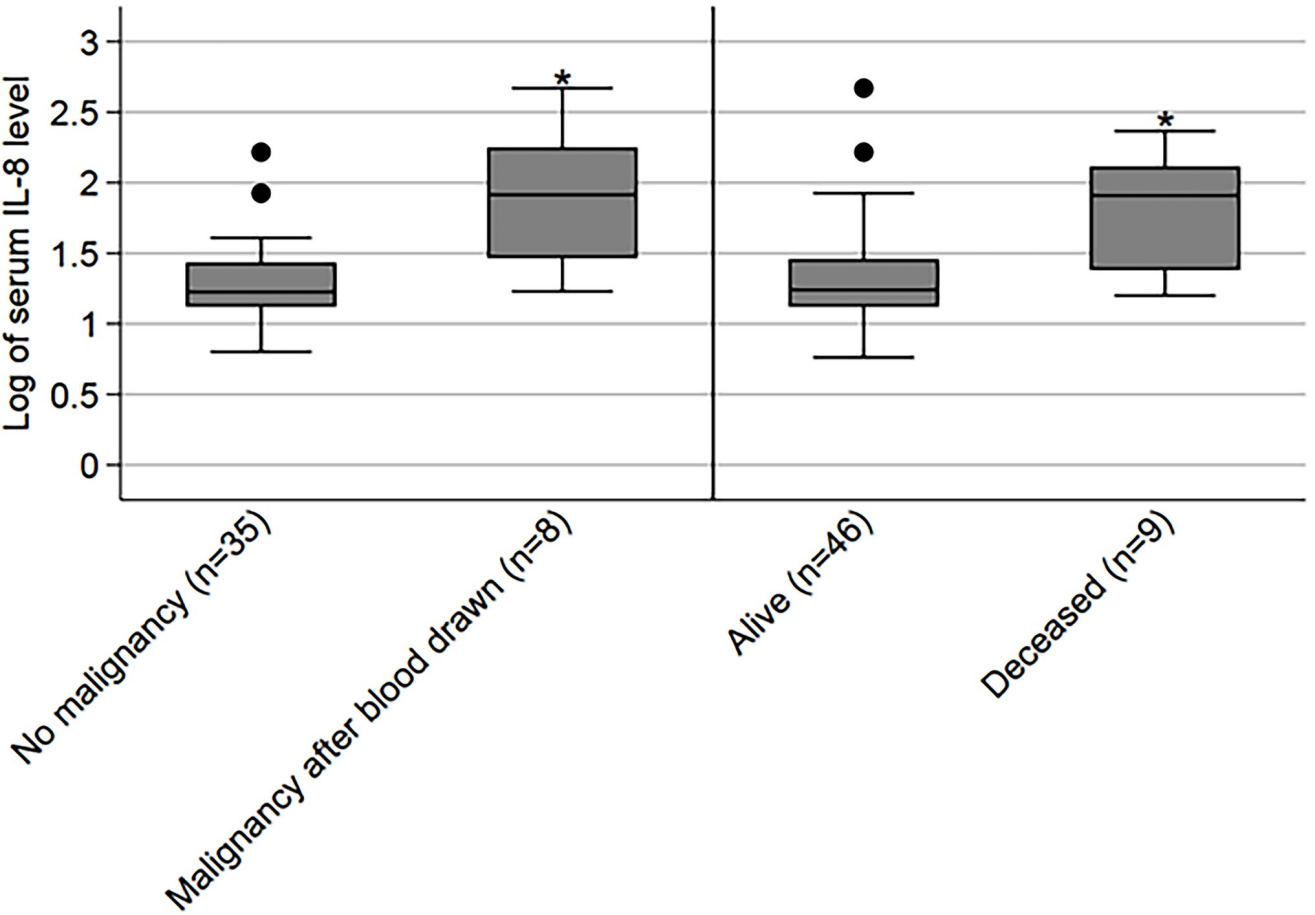
Left pair of box plots demonstrate median and interquartile ranges for log transformed serum IL-8 levels for subjects who developed and did not develop malignancy following specimen collection; subjects who had developed malignancies prior to specimen collection were excluded from this pair of plots. The right pair of box plots depicts similar data for subjects alive at the end of the follow-up period compared to those who died. *Regression *p* values adjusted for age, sex, and IL6 were 0.009 for any malignancy and 0.034 for death.

Several processes associated with inflammation (TNFα, NF-κβ inflammatory response) and malignancy (MTORC1) were enriched by transcriptional profiling in subjects with classic A-T. Since transcriptional regulation of IL8 expression has been shown to be regulated through NF-κβ activation, [30] we examined long-term outcomes of subjects with A-T who previously had baseline IL8 and IL6 levels measured. [11] This was done to determine if an association existed between higher levels of baseline serum IL8 and IL6 and the development of a malignancy and/or death at 4–6 years of follow-up (Table 1). After adjusting for sex and age, log of serum IL8 levels was associated with an increased risk of malignancy (*p = 0*.*009*) and death (*p = 0*.*034*), within the follow-up period, in logistic regression models, (Fig 5). In a combined model with log of serum levels of both IL6 and IL8, IL6 was not associated with malignancy (*p = 0*.*96*) or death (*p = 0*.*46*). Sex was an independent predictor of death and was not based on age or IL8 levels.

**Table 1:** Association between serum IL8 and IL6 levels and subsequent clinical diagnosis of malignancy and/or death over a 4–6 year follow-up period*.

Mean ± S.D [Range]	No Malignancy in Follow-up Period (n = 39)	Malignancy in Follow-up Period (n = 9)	*P* Value	Alive in Follow-up Period (n = 51)	Deceased in Follow-up Period (n = 10)	*P* Value
**Age at time of specimen (**years)	12.2 ± 7.6 [1, 27]	16.8 ± 2.3 [8, 25]	0.11	12.7 ± 7.6 [1, 27]	18.4 ± 7.1 [7, 26]	**0.031**
**Sex** (% female)	59.0	44.4	0.42	54.9	20.0	**0.044**
**Log Serum IL6** (log pg/mL)	0.30 ± 0.45 [-0.32, 1.56]	0.64 ± 0.49 [-0.05, 1.41]	0.051	0.37 ± 0.46 [-0.32, 1.56]	0.68 ± 0.34 [-0.05, 1.02]	**0.045**
**Log Serum IL8** (log pg/mL)	1.28 ± 0.27 [0.80, 2.22] (n = 35)	1.90 ± 0.51 [1.23, 2.67] (n = 8)	**< 0.001**	1.31 ± 0.34 [0.76, 2.67] (n = 46)	1.74 ± 0.44 [1.20, 2.37] (n = 9)	**0.002**

*After adjusting for sex and age, in combined models with both IL6 and IL8, log of serum IL8 levels was associated with an increased risk of malignancy (p = 0.009) and death (p = 0.034) within the follow-up period, but log of serum IL6 levels was not associated with malignancy (p = 0.96) or death (p = 0.46).

Taken together, these findings indicate that transcriptional profiling in subjects with classic A-T aligns with markers of systemic inflammation and malignancy risk/death in people with A-T.

### Validation of gene expression in a separate cohort of subjects with classic A-T

From the RNAseq data, we randomly selected four representative DEGs for validation by RT- qPCR. These genes included, ADAM23, NRCAM, CNTNAP2/ CASPR2 and LTBP1. Although these genes were randomly selected from the larger number of differentially expressed genes, they all had functions that could be relevant to the A-T phenotype. Expression of ADAM23 has been shown on T cells and dendritic cells (DCs) [31] and downregulation of ADAM23 on DCs can impair Th differentiation and cell proliferation in OVAp-specific CD4^+^ T.[31] In addition, NRCAM is an adhesion gene and altered expression of NRCAM has been associated with certain malignancies. [32] [33] [34] CNTNAP2/CASPR2 is a member of the neurexin family, and dysregulation of CNTNAP2 has been associated with neurologic symptoms and language impairment such as dysarthria. [35] Serum antibodies to CNTNAP2/CASPR2 have been reported in patients with progressive cerebellar ataxia.[36]

These genes were validated in three separate cohorts of patients. Specimens were obtained from subjects with classic A-T phenotypes, mild A-T phenotypes and non-A-T healthy controls. Classic and mild phenotypes were determined by clinical and laboratory characteristics, (Table 2). The subjects with mild A-T were significantly older, had lower serum α-fetoprotein, and were more likely to have normal immune function and ATM mutations predicting some ATM protein production compared to classic A-T subjects. The non-A-T healthy controls were significantly older compared to classic A-T subjects. All subjects were recruited prospectively.

**Table 2:** Clinical and laboratory characteristics of subjects with Classic A-T phenotypes and Mild A-T phenotypes.

Mean (± SD) [Range]	Mild Ataxia telangiectasia (n = 8)	Classic Ataxia telangiectasia (n = 7)	T Test *P* Value
**Age (years)**	19.12 ± 10.7 [4–39]	9.43 ± 4.3 [3–13]	**<0.044**
**Sex (% female)**	50%	42.8%	<0.800
**Low IgA and/or low IgG and/or need for supplemental gamma globulin**	0% (n = 8)	85.7% (n = 7)	**<0.00002**
**Serum α-fetoprotein (ng/ml)**	49.3 ± 59.7 (n = 6)	130.1 ± 76.8 (n = 7)	**<0.053**
**% Force vital capacity**	74% ± 15.5 (n = 7)	58% ± 13.9 (n = 5)	<0.094
**Predicted partial ATM protein production based on ATM mutation**	87.5% (n = 8)	0% (n = 5)	**0.0002**

**Fig 6:**
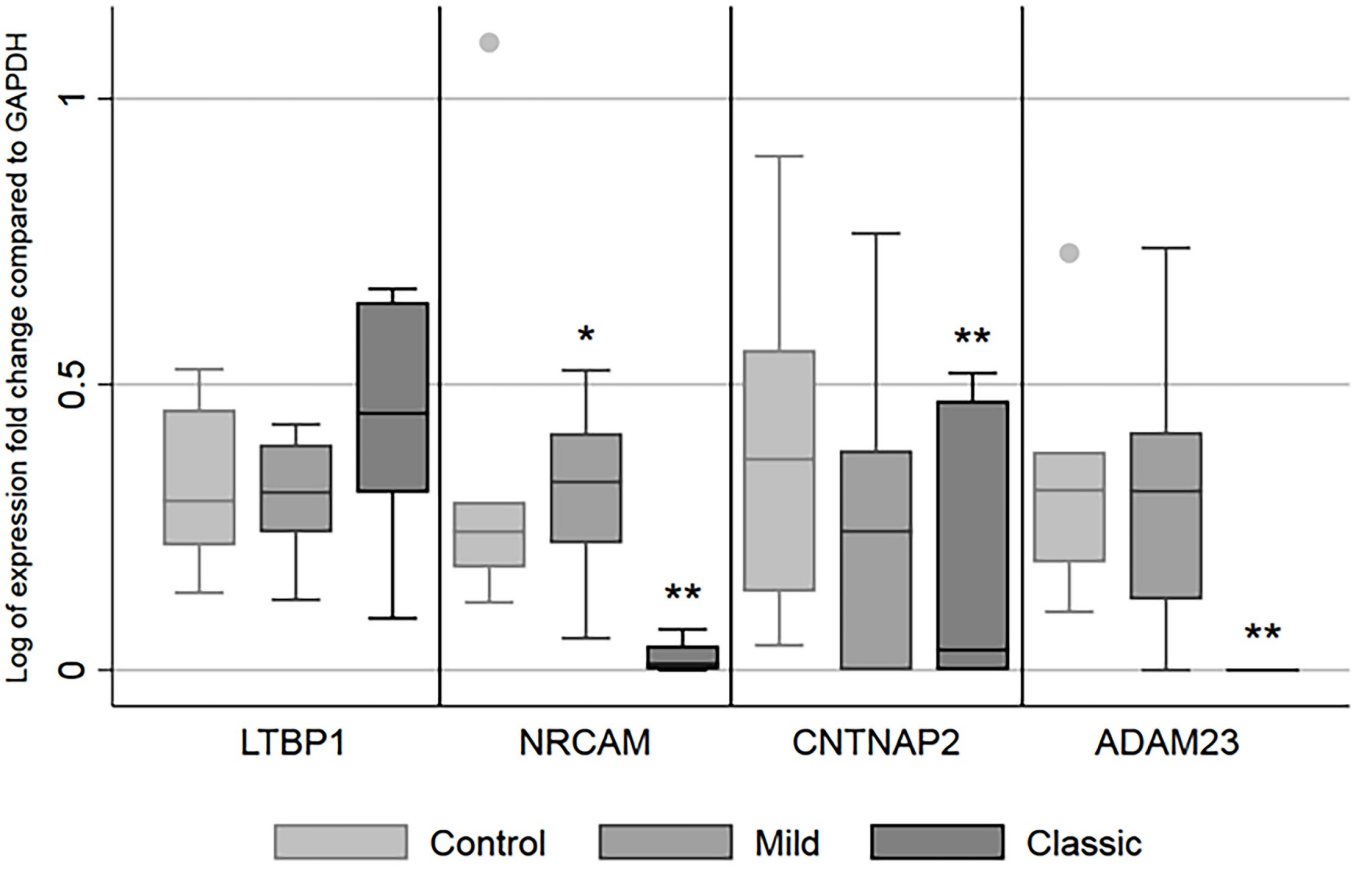
Four differentially expressed genes (DEGs) from RNAseq data validated by RT-PCR in subjects recruited prospectively. Box plots of selected DEGs by clinical group (non-A-T healthy controls, Mild A-T phenotype and Classic A-T phenotype). *Adjusted *p*<0.05 for Mild A-T phenotype compared to non-A-T healthy controls. **Adjusted *p*<0.05 for Classic A-T phenotype compared to non-A-T healthy controls. RNA was isolated from PBMCs obtained from Classic A-T (n = 7), Mild A-T (n = 8) and non-A-T healthy controls (n = 6). *P* values were derived through linear regression and adjusted for age.

RT- qPCR revealed that three of four genes initially selected for validation were significantly downregulated in subjects with classic A-T compared to non-A-T healthy controls. Though trending towards higher expression, the fourth gene LTBP1, predicted to be upregulated in the classic A-T cohort, did not reach significance by RT- qPCR. Of the genes that were validated, expression of ADAM23, was undetectable in PBMCs in subjects with classic A-T. (Fig 6) When adjusted for age, the subjects with classic A-T had significantly lower expression of ADAM23, NRCAM and CNTNAP2 in PBMCs when compared to non-A-T healthy controls. Subjects with mild A-T phenotypes also had significantly higher expression of NRCAM compared to classic A-T subjects. Older age was significantly associated with decreased NRCAM expression (p<0.003). When using age-adjusted regression, the log of expression fold change for mild and classic A-T phenotype was less than control by -0.23 and -0.68 respectively. Following this, we selected several other genes that were upregulated by RNAseq for validation by RT-qPCR using RNA from PBMCs from three young subjects with A-T (mean age 9 years ±1). We found that integrin β5 (ITGB5) and TAL1 expression was significantly increased in the PBMCs of these A-T subjects compared to non- A-T controls (1.86 versus 1.08, *p<0*.*035* and 1.94 and 1.09, *p< 0*.*029* respectively). ITGB5 has been shown to contribute to tumor development in breast cancer, epithelial-mesenchymal transition and tumor potential in carcinoma cells [37, 38] and TAL1 is T-cell pro-oncogene with over-expression associated with development of leukemia. [39] ALOX12, CLU and ADRA2A were also upregulated in the A-T subjects but did not reach statistical difference compared to non A-T controls.

## Discussion

Using RNAseq, we identified 310 differentially expressed genes from PBMCs of subjects with classic A-T phenotypes. We found that over half of the variance in our study, was primarily driven by the classic A-T phenotype. Using hallmark gene sets [25], A-T subjects were found to have processes enriched for inflammation, malignancy and cell growth. Based on these results we examined a separate cohort of A-T subjects who had baseline serum IL6 and IL8 measured previously to determine if an association existed between higher serum IL6 and IL8 levels and subsequent development of malignancy and/or death. During a 4 to 6 year follow-up period, we found that serum IL8 levels were associated with an increased risk of malignancy (*p = 0*.*009*) and death (*p = 0*.*034*) using logistic regression models. These findings support an association between inflammation and malignancy in people with A-T and suggest that RNAseq signatures could be used as biomarkers in identifying people at increased risk for A-T related morbidities and to direct therapy to mitigate disease causing phenotypes.

Using RNAseq, we found that PBMCs from subjects with classic A-T had altered expression of genes involved in regulation of inflammation and immunity. Dysregulation of these gene pathways may contribute to the inflammatory and immune abnormalities frequently found in people with classic A-T. In particular, we found that A-T PBMCs exhibited downregulation of immune response genes involved in B cell development (DTX1), B cell migration (CXCR5) and B cell differentiation (FCER2) compared to non-A-T PBMCs. In addition, altered expression of immune response genes involved in T cell responses and inflammation were also found. CD200 an immune checkpoint regulator that regulates inflammation, including IL6 expression [40] was downregulated in subjects with classic A-T. Taken together these findings indicate a relationship between immune dysregulation and inflammation in A-T.

Immune dysregulation can be a factor in the development of cancer. We found that ADAM23 and BACH2 were downregulated by RNAseq in subjects with classic A-T. Both of these genes have roles in T cell regulation and tumor suppressor (TS) functions. BACH2 haplo-insufficiency has been associated with lymphocyte-maturation defects leading to immunoglobulin deficiency and intestinal inflammation. [41] ADAM23 has been shown to regulate T cell proliferation and effector T cell function. Knockdown of ADAM23 in bone marrow-derived dendritic and splenic cells can impair CD4^+^ T cells effector responses and decrease T cell proliferation. [31] Our studies suggest that downregulation of ADAM23 and BACH2 may contribute to the altered T cell immune responses and increased cancer risk found in people with classic A-T.

The use of transcriptional profiling has allowed for the identification of novel disease markers and molecular targets in many human diseases. [42] Recently others, have used microarrays to demonstrate gene expression changes in A-T cells treated with dexamethasone (DEX). These researchers found altered gene expression in peripheral blood mononuclear cells isolated from A-T subjects, who received DEX compared to controls and were able to demonstrate gene expression differences in DEX treated A-T lymphoblastoid cells lines.[43, 44] These studies indicate that transcriptional profiling could be used in people with A-T to identify responses to therapy through changes in biological markers.

There are several limitations to this study. We used PBMCs from subjects with A-T and compared them to non-A-T healthy controls. RNAseq in subjects with lymphopenia could result in an under-representation of peripheral lymphocyte gene expression. To address this, we normalized samples using Ficoll-Paque Plus to enrich for lymphocytes. Another potential limitation to our study is the inherent variability in A-T with regard to systemic inflammation, cancer development and immune dysregulation, making it less likely to identify biological pathways that are consistently dysregulated in all/most people with A-T. This variability in A-T phenotype is likely due to the lack of mutational hotspots on the ATM gene, diverse environmental exposures, age effects and nutritional status differences. Despite these barriers, we were able to identify specific genes and biological processes dysregulated in our three subjects with classic A-T using RNAseq. Although, a larger sample size for the RNAseq would minimize variation and bias, we were able to validate several DEGs found by RNAseq, using RT-qPCR in a separate cohort of subjects with classic A-T, expanding generalizability of these findings. We expect however that people with mild A-T phenotypes, will have a more variable or distinct RNAseq molecular signature compared to people with classic A-T. Another limitation to our study was that temporal variability in PBMC transcriptional profiles was not assessed by longitudinal sampling of study participants. Our subjects however were study at baseline, were not acutely ill and our aim was to understand differences between AT patients and controls in the steady state and thus biological replicates were not pursued. We also only obtained one sample per individual (technical replicate) at a time. This could be considered a limitation, however technical replicates (as opposed to biological replicates) are rarely performed in RNA-sequencing experiments due to the very high technical reproducibility of the technique without additional gains in power. [45] Despite these limitations, our results indicate that RNAseq signatures could potentially be used to differentiate disease risk with regard to inflammation and immune dysregulation in people with A-T. Future studies are needed to validate additional DEGs and biological processes dysregulated in PBMCs of people with A-T and to assess longitudinal variability over time.

In summary, we identified DEGS and inflammatory biological processes that were dysregulated in subjects with classic A-T. These DEGS included BACH2, a gene involved in T cell regulation and tumor suppression. Use of RNAseq also allowed us to identify gene sets enriched in A-T subjects that were associated with inflammation, cancer, and immune abnormalities. We also demonstrated a positive association between higher baseline serum IL8 levels and a subsequent diagnosis of malignancy and/or death. Use of PBMCs to identify DEGs and biological processes altered in people with A-T could be used to differentiate disease risk and severity in individuals with A-T. Furthermore, identification of specific inflammatory pathways dysregulated in A-T individuals, could be potentially targeted to attenuate inflammation and modify cancer risk.

## Supporting information

S1 Fig Enrichment plots for the top 15 hallmark gene sets. (TIF)

S1 Table Rsem expected counts for the RNA-sequencing data. [provided as a tab-delimited file].
(TXT)
