# Supplementary figures and images for: Inflammation and transcriptional responses of peripheral blood mononuclear cells in classic ataxia telangiectasia
Source: PLoS One. 2018 Dec 26;13(12):e0209496. doi: 10.1371/journal.pone.0209496 (PMC6306200; doi:10.1371/journal.pone.0209496)

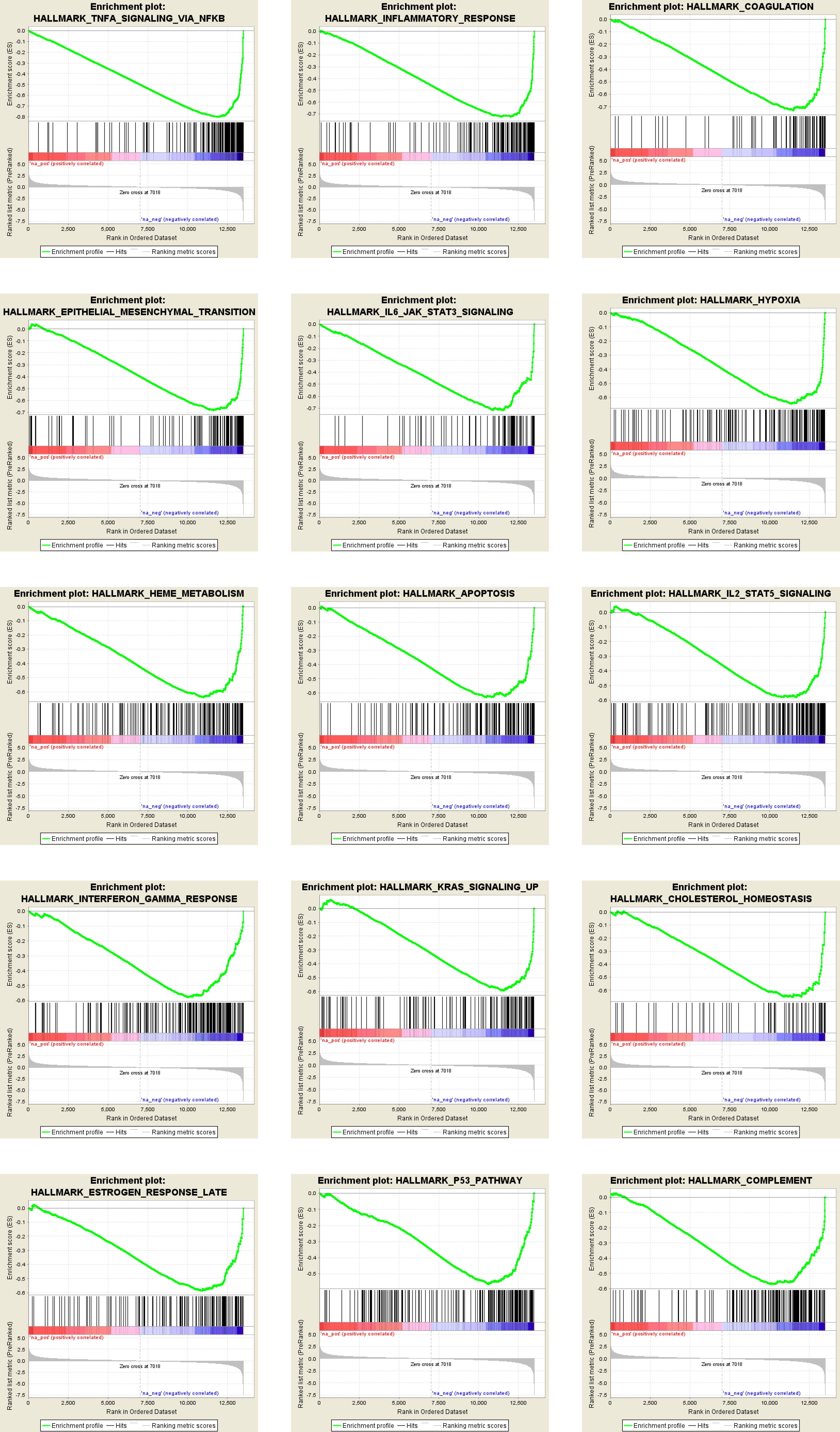

Supplement: S1 Fig — (TIF) [file pone.0209496.s001.tif]
